# Nationwide Hospital-Based Survey of Adult T-Cell Leukemia/Lymphoma in Japan

**DOI:** 10.3390/v14040791

**Published:** 2022-04-11

**Authors:** Takeharu Kato, Yoshitaka Imaizumi, Yasushi Miyazaki

**Affiliations:** 1 Department of Hematology, Nagasaki University Hospital, Nagasaki 852-8501, Japan; 2 Department of Hematology, Atomic Bomb Disease and Hibakusha Medicine Unit, Atomic Bomb Disease Institute, Nagasaki University, Nagasaki 852-8501, Japan; y-imaizm@nagasaki-u.ac.jp (Y.I.); y-miyaza@nagasaki-u.ac.jp (Y.M.)

**Keywords:** adult T-cell leukemia/lymphoma, clinical subtype, prognosis, nationwide survey

## Abstract

Nationwide surveys of adult T-cell leukemia/lymphoma (ATL) have played an important role in helping us to understand the pathophysiology of this disease and analyze its prognosis in Japan. Classifications of clinical subtypes have been proposed based on the results of nationwide surveys of patients with ATL diagnosed in the 1980s. This article highlighted the classification and prognosis of ATL based on different surveys and focused on the comparison of data derived from the available surveys. The 11th nationwide hospital-based survey was conducted in patients with ATL diagnosed in 2010–2011 using the same method as that used in the 1980s survey. The median age of disease onset was 68 years, which was increased compared with previous surveys. While median survival of patients with the acute and lymphoma types had not improved much since the 1980s, the 4-year survival rate was higher. Little improvement in the prognosis was observed for the chronic and smoldering types. The 12th nationwide survey of patients with ATL diagnosed in 2012–2013 also showed an increase in age at onset. Further epidemiological research that includes more cases is needed to deepen our understanding of the actual state of treatment and prognosis of this disease.

## 1. Introduction

Adult T-cell leukemia/lymphoma (ATL) is a disease in which mature T-cells infected with human T-cell leukemia virus type I (HTLV-1) form tumors [1,2]. HTLV-1 is endemic in Japan, Latin America, southwest Africa, and parts of Australia, and these countries have many patients with ATL [3]. In Japan, the nationwide estimation of the number of HTLV-1 carriers was at least 1.08 million and the lifetime risk of ATL among HTLV-1 carriers was estimated to be 4–6% for men and 2.6% for women in several HTLV-1-endemic areas [4]. The incidence of ATL in Japan is estimated to be 1000–1500 per year, making it a rare hematologic malignancy [4]. Therefore, large-scale analyses based on nationwide surveys have played an important role in understanding the prognosis and pathophysiology of ATL in Japan. This review describes the results of these nationwide surveys, focusing on more recent reports.

## 2. Adult T-Cell Leukemia/Lymphoma

In 1977, Uchiyama and Takatsuki et al., of Kyoto University published the first report on the clinical and hematological features of adult T-cell leukemia based on 16 cases with this disease [5]. They reported that the typical clinical and hematological features included adult onset, subacute or chronic leukemia with a rapidly progressive terminal course, leukemia cells often characterized morphologically by deeply indented or lobulated nuclei, frequent skin involvement, and often lymphadenopathy and hepatosplenomegaly. They also reported one particular epidemiological characteristic, namely, that most patients were from the Kyushu region, and mentioned the possibility of a viral cause. In 1981, Hinuma et al., of Kyoto University examined ATL-derived cultured cells and serum from patients with ATL. They detected cells positive for ATL-associated antigen (ATLA) in cells cultured with the indirect fluorescent antibody technique and found type C retrovirus particles in cultured cells using an electron microscope [6]. In their study, patients with ATL were found to be positive for ATLA antibodies (anti-HTLV-1 antibodies), demonstrating the usefulness of ATLA antibodies for diagnosing ATL.

## 3. Classifications of ATL Type

With advances in the methods used to diagnose ATL, various clinical and pathological conditions have been reported in addition to the initial reports of a poor prognosis with rapid progression and leukemic features. Classifications were proposed based on various subtypes in addition to the acute type originally reported by Uchiyama et al. [5], including a lymphoma type that primarily features lymph node lesions without prominent increase in abnormal lymphocytes in the peripheral blood, a chronic type that exhibits leukemic features but progresses chronically, and a smoldering (or pre-ATL) type with a small number of abnormal cells in the peripheral blood and skin/lung lesions that progress slowly [7,8,9]. However, there are still no clear diagnostic criteria for each subtype, which makes it difficult to determine the subtype when diagnosing ATL. The third and fourth nationwide ATL hospital-based surveys were conducted by Shimoyama et al., from the National Cancer Center Japan in a large number of patients with ATL diagnosed in 1983–1987. Based on an analysis of 818 eligible cases, diagnostic criteria for clinical subtypes (i.e., the Shimoyama classification) were proposed [10]. This classification system includes four subtypes of ATL, based on the clinical findings at the time of diagnosis: acute, lymphoma, chronic, and smoldering types. Median survival was 6.2 months for the acute type, 10.2 months for the lymphoma type, 24.3 months for the chronic type, and not yet reached for the smoldering type, confirming that the prognosis could be stratified based on the clinical subtype (1991 report, Table 1). Furthermore, lactate dehydrogenase, blood urea nitrogen, and albumin were found to be independent prognostic factors for chronic ATL. Among the cases of chronic ATL, those with normal lactate dehydrogenase, blood urea nitrogen, and albumin levels showed slow progress, similar to that of the smoldering type, while those in which any of these factors were abnormal had a poor prognosis [11]. Based on these findings, the development of treatments for ATL has been based on these clinical subtypes and prognostic factors, and clinical studies have been conducted for the acute, lymphoma, and chronic types with poor prognostic factors (unfavorable chronic type) [12,13,14]. The current ATL treatment algorithm used in Japan follows these criteria [15]. When ATL is diagnosed, the disease subtype is determined and combination chemotherapy is administered for the acute, lymphoma, and unfavorable chronic types. Thereafter, allogeneic hematopoietic stem cell transplantation (allo-HSCT) can be considered, if indicated. Patients with the smoldering or chronic type without poor prognostic factors (favorable chronic type) are followed up on without treatment until the disease progresses or treated for any skin lesions.

## 4. Prognosis of ATL in the 2000s

Katsuya and Ishizuka, then at Fukuoka University, conducted a nationwide retrospective survey of patients with ATL diagnosed in 2000–2009 to clarify the prognosis of cases detected more recently, with the goal of developing prognostic indicators [16]. This study retrospectively examined 1594 cases, which is the largest number to date. Median survival was 8.3 months for the acute type, 10.6 months for the lymphoma type, 31.5 months for the chronic type, and 55.0 months for the smoldering type (2015 report, Table 1). Furthermore, regarding the prognosis of the chronic type, median survival was 27 months when poor prognostic factors were present and not yet reached when they were not present (Table 1). The Shimoyama classification and the chronic-type prognostic factors were also useful for stratifying the prognosis in cases of ATL diagnosed in the 2000s. Although the prognostic pattern for each of the subtypes was similar to that in the 1980s, 4-year overall survival had improved slightly in the acute and lymphoma subtypes. In contrast, the prognoses of the smoldering and chronic types had improved little.

### 4.1. Acute Type and Lymphoma Type

Katsuya et al., analyzed the prognostic factors for the acute and lymphoma types and for the chronic and smoldering types based on their own data [17,18]. The prognostic factors extracted for the acute and lymphoma types were age, clinical stage, performance status, and serum albumin and soluble interleukin-2 receptor (sIL2R) levels. Based on these factors, they developed two prognostic indices (PIs), namely an ATL-PI and a simplified ATL-PI (Table 2). When cases were separated based on the number of poor prognostic factors into low-risk, intermediate-risk, and high-risk groups, the median survival durations were 4.6, 7.0, and 16.2 months, respectively, enabling stratification of the prognosis into three groups using the simplified ATL-PI. However, the prognosis could not be considered good even in the low-risk group; therefore, this index is not considered clinically applicable for stratifying cases prospectively before deciding on the treatment strategy.

### 4.2. Smoldering Type and Chronic Type

Prognostic factor analysis was also performed for the smoldering and chronic types, which extracted sIL2R as an independent prognostic factor [18]. An indolent ATL-PI and a simplified ATL-PI were developed based on this finding. When cases were divided into low-risk, intermediate-risk, and high-risk groups based on the sIL2R value, the prognosis could be stratified into three groups, that is, not yet reached, 5.5 years, and 1.6 years, respectively (Table 3). In an analysis of the time to systemic chemotherapy using the conventional subtype classification and poor prognostic factors, the median time to systemic chemotherapy for chronic cases with poor prognostic factors was not good at 0.3 years, while those for smoldering cases and chronic cases without poor prognostic factors were similar at 4.5 and 5.1 years, respectively. Among chronic cases with poor prognostic factors, the rate of cumulative chemotherapy in year 1 was about 60% and about 80% even in year 4; hence, some cases survived without receiving chemotherapy. The fact that these patients with slow progression were included in the unfavorable chronic type suggests that the conventional subtypes and prognostic factors cannot adequately identify these slowly progressing cases. Meanwhile, when time to systemic chemotherapy was examined by indolent ATL-PI risk stratification, the median time to systemic chemotherapy was 8.4 years in the low-risk group, 2.7 years in the intermediate-risk group, and 0.1 years in the high-risk group, indicating that three-group stratification was possible. Based on this finding, Katsuya et al., proposed follow-up with no treatment for the low-risk group, chemotherapy or combination therapy with interferon alfa and zidovudine for the high-risk group, and a clinical study for the intermediate-risk group. However, this proposal has not been validated in a large cohort, and the issue of how the intermediate group should be treated in clinical practice remains unresolved.

## 5. Eleventh Nationwide Hospital-Based Survey

The 11th nationwide hospital-based survey was conducted in patients with ATL newly diagnosed in 2010–2011. Data for this survey were collected in the same manner as for the nationwide survey in the 1980s. Data for 996 cases from 173 institutions were collected, and 922 eligible cases were analyzed [19]. The median age at onset was 68 years (mean 67.5 years), which was higher than in previous surveys (Table 4). Furthermore, only 5.6% were younger than 50 years of age at onset, which represented a marked decline. An examination of subtype and age at diagnosis showed a higher proportion of lymphoma types among the elderly. An examination of changes in the distribution of subtypes over time showed a decrease in the acute type and an increase in the smoldering and chronic types compared with past surveys (Table 4).

## 6. Study of Prognosis in the 11th Nationwide Hospital-Based Survey

The prognosis was investigated in 770 eligible cases in the 11th survey [20]. The distribution by subtype was 391 acute cases, 192 lymphoma cases, 106 chronic cases, and 81 smoldering cases. The median survival time was 8.3 months for the acute type, 10.0 months for the lymphoma type, 25.6 months for the chronic type, and 60.9 months for the smoldering type (Table 1). The prognostic pattern for each type of ATL was similar to that in the 1980s, with the acute and lymphoma types progressing rapidly with a poor prognosis and the chronic and smoldering types progressing relatively slowly.

### 6.1. Acute and Lymphoma Types

Compared with the studies by Shimoyama et al. [10] and Katsuya et al. [11], there was little improvement in median survival time in patients with acute type or lymphoma type ATL. Nevertheless, compared with the 1991 report by Shimoyama et al., 4-year survival showed gradual improvement in the 2015 report by Katsuya et al., and in the present 2020 report (Table 1). When the prognosis of cases with acute-type or lymphoma-type ATL diagnosed before the age of 70 years was examined based on whether allo-HSCT was performed, the 4-year survival rate was 38.9% for the acute type and 39.1% for the lymphoma type among those who underwent transplantation, indicating that this can be expected to improve the long-term prognosis. In contrast, the 4-year survival rate of patients who did not undergo transplantation was 17.0% for the acute type and 26.3% for the lymphoma type, indicating a particularly poor prognosis for the acute type (Table 5). In acute ATL, given that about 58% of all patients were under 70 years of age and 31% of those patients underwent transplantation, it is estimated that about 18% of all acute ATL cases undergo transplantation. To improve the overall prognosis of acute ATL by transplantation, improvement is needed in both transplant outcomes and the percentage of patients receiving transplants in the 2021 report, which were similar to that in the 2015 report. Furthermore, because a large proportion of patients die without receiving a transplant, it is important to develop treatments for patients for whom transplantation is not indicated.

### 6.2. Chronic and Smoldering Types

Median survival was 1.6 years in patients with the unfavorable chronic type and 5.3 years in those with the favorable chronic type, which indicates that the prognosis is significantly poorer in patients with poor prognostic factors established in previous studies. Compared with the report for the patients diagnosed in the 1980s by Shimoyama et al. [10], the 4-year survival rate of the chronic type improved slightly, and that of the smoldering type did not improve at all in either the report by Katsuya et al. [16] or the present study (Table 1). Analysis of smoldering ATL by presence or absence of skin lesions showed that cases with skin lesions tended to have poorer survival, but this finding was not significant (Table 6).

## 7. The 12th Survey

Following the 11th nationwide hospital-based survey, a 12th survey was performed in patients with ATL diagnosed nationwide in 2012–2013 with the aim of collecting data on a large number of cases by extracting candidate patients using four existing registries. Ultimately, data on 1042 cases were collected from 117 institutions, and 984 eligible cases were analyzed [21]. In this study, the median age at onset was 69 years and the mean was 67.9 years, which is similar to the results of the 11th survey (Table 4). The proportions of each subtype were broadly similar to those of the 11th survey. Examination of subtype by age at diagnosis confirmed an increasing proportion of the lymphoma subtype in elderly patients, which again is similar to the results of the 11th survey (Table 7). An examination of the relationship between diagnosis site and home region showed that about 30–60% of urban cases (diagnosed in Kanto, Chubu, Kinki) were originally from Kyushu, which is an endemic area. This suggests that a percentage of urban patients had moved from Kyushu. Furthermore, examination of the relationship between diagnosis site and age at onset showed that at least 60% (61.0–63.5%) of cases in urban areas (Kanto, Chubu, Kinki) were aged 69 or younger, which is the overall median age, while in Kyushu, the proportion was smaller at 38.7%, indicating more pronounced patient aging (Table 8).

## 8. Conclusions

A large number of institutions have participated in nationwide ATL hospital-based surveys, which have had an important role in understanding the actual state of treatment of ATL and in creating treatment strategies. ATL is a rare disease, and nationwide surveys play a major role in analyzing large numbers of cases. Recent reports have shown an increase in the age at onset of ATL. The prognosis of the acute and lymphoma types has improved slightly, but the situation is still not satisfactory. The prognosis of the chronic and smoldering types has not improved since the 1980s, and treatment strategies need to be reexamined. Treatment options for ATL have increased in recent years. For allo-HSCT, the range of indications has expanded with the introduction of haploidentical transplants. New drugs have also been introduced for preventing and treating infectious diseases. Therefore, there is a need for further studies on the recent implementation status of allogeneic transplants and the prognosis of the patients treated with transplantation. Furthermore, several new agents for ATL have become available in Japan. Mogamulizumab was the first to be approved in 2012, followed by lenalidomide, brentuximab vedotin, and tucidinostat [22,23,24,25,26]. Many of the patients whose prognosis was examined in the 11th survey had not taken these drugs. Therefore, the effects of these agents on the prognosis of ATL constitute an important topic for future surveys. To evaluate the actual state of treatment and prognosis of ATL, further studies suited to various objectives are needed going forward.

## Figures and Tables

**Table 1:** The distribution, age at diagnosis, and prognosis of ATL by clinical subtypes.

	Clinical Subtype	1991 Report (1983–1987)	2015 Report (2000–2009)	2021 Report (2010–2011)
Total No. of patients		818	1594	770
Percentage of patients (%)	Acute	56.8	56.2	50.8
	Lymphoma	19.1	22.3	24.9
	Chronic	18.6	11.7	13.7
	Smoldering	5.5	9.8	10.5
Age at diagnosis (Mean/Median)	Acute	56.0/NA	63.0/63.0	NA/68.0
	Lymphoma	59.2/NA	66.0/66.0	NA/70.0
	Chronic	57.7/NA	61.0/61.0	NA/65.0
	Smoldering	59.3/NA	67.0/67.0	NA/68.0
Median survival time (months)	Acute	6.2	8.3	8.3
	Lymphoma	10.2	10.6	10.0
	Chronic	24.3	31.5	25.5
	Favorable	NA	Not reached	63.5
	Unfavorable	NA	27.0	18.8
	Smoldering	Not reached	55.0	60.7
4-year overall survival rate (%)	Acute	5.0	11.4	16.8
	Lymphoma	5.7	16.2	19.5
	Chronic	26.9	35.6	34.7
	Favorable	NA	60	62.1
	Unfavorable	NA	29	26.6
	Smoldering	62.8	52	59.8

ATL, adult T-cell leukemia-lymphoma; NA, not available.

**Table 2:** Simplified ATL-PI.

Prognostic Factors			Score
Stage III–IV			2
ECOG PS 2–4			1
Age, years > 70			1
Serum albumin, g/dL < 3.5			1
sIL2R, U/mL > 20,000			1
**Risk Group**	**Score**	**Median OS (Year)**	**2-Year OS Rates (%)**
Low	0–2	16.2	37
Intermediate	3–4	7.0	17
High	5–6	4.6	6

ATL-PI, a prognostic index (PI) for acute- and lymphoma-type adult T-cell leukemia/lymphoma (ATL); PS, performance status; OS, overall survival; sIL-2R, soluble interleukin-2 receptor.

**Table 3:** Indolent ATL-PI (iATL-PI).

Simplified iATL-PI Risk Group	Sil-2R (U/Ml)	Overall Survival Median (Year)	Time to Systemic Chemotherapy Median (Year)
Low	<1000	Not reached	8.4
Intermediate	≥1000 and 6000≤	5.5	2.7
High	>6000	1.6	0.1

ATL, adult T-cell leukemia/lymphoma; iATL-PI, a prognostic index (PI) for chronic- and smoldering-type ATL (indolent ATL); sIL-2R, soluble interleukin-2 receptor.

**Table 4:** Comparison of the results of nationwide surveys.

Year of Diagnosis of Patients	1992–1993	1994–1995	2006–2007	2010–2011 (11th Survey)	2012–2013 (12th Survey)
Total No. of patients	712	753	910	922	984
Age at diagnosis, year					
median	NA	NA	67	68	69
mean	58.9	60.3	66	67.5	67.9
Subtype					
Acute	489 (69.4%)	423 (57.6%)	46.7%	456 (49.5%)	511 (51.9%)
Lymphoma	151 (21.4%)	176 (24.0%)	34.8%	237 (25.7%)	245 (24.9%)
Chronic	36 (5.1%)	95 (12.9%)	8.2%	131 (14.2%)	123 (12.5%)
Smoldering	29 (4.1%)	40 (5.4%)	10.3%	98 (10.6%)	105 (10.7%)

NA, not available.

**Table 5:** Survival of the patients treated with or without allogeneic hematopoietic cell transplantation (Allo-HSCT).

Subtype	Allo-HSCT	Patient <70 Years	1991 Report (1983–1987)			2015 Report (2000–2009)			2021 Report (2010–2011)		
			*N*	Median OS (Months)	4-Year OS Rate (%)	*N* (%)	Median OS (Months)	4-Year OS Rate (%)	*N* (%)	Median OS (Months)	4-Year OS Rate (%)
Acute	Yes	<70	0	NA	NA	178 (20)	14.0	27.8	61 (18)	19.2	38.9
	No		465	6.2	5.0	717 (80)	6.7	6.8	148 (40)	9.2	17.0
		≧70							NA (42)	NA	7.2
Lymphoma	Yes	<70	0	NA	NA	49 (14)	13.9	32.3	20 (11)	14.3	39.1
	No		156	10.2	5.7	306 (84)	9.7	13.7	65 (38)	9.7	26.3
		≧70							NA (51)	NA	11.3

OS, overall survival; NA, not available.

**Table 6:** Survival of the patients with smoldering ATL positive or negative for skin lesions.

Skin Lesion	Median OS (Months)	4-Year OS Rate (%)
Positive	57.2	54.3
Negative	Not reached	68.5

ATL, adult T-cell leukemia/lymphoma; OS, overall survival.

**Table 7:** Age at diagnosis and clinical subtypes.

Age at Diagnosis	Total	<50	50–59	60–69	70–79	≥80
Subtype	(%)	(%)	(%)	(%)	(%)	(%)
Acute	52	49	61	52	49	49
Lymphoma	25	13	16	21	32	32
Chronic	13	27	11	16	11	5
Smoldering	11	12	13	10	8	14

**Table 8:** Age at diagnosis and area of diagnosis.

Age at Diagnosis	Hokkaido (*N* = 32)	Tohoku (*N* = 26)	Kanto (*N* = 52)	Chubu (*N* = 41)	Kinki (*N* = 95)	Chugoku/Shikoku (*N* = 56)	Kyushu (*N* = 727)
≥69(%)	46.9	42.3	36.5	39.0	36.8	51.8	61.3
<69(%)	53.1	57.7	63.5	61.0	63.2	48.2	38.7

## Data Availability

Not applicable.
